# Utility of Three Adiposity Indices for Identifying Left Ventricular Hypertrophy and Geometric Remodeling in Chinese Children

**DOI:** 10.3389/fendo.2021.762250

**Published:** 2021-11-16

**Authors:** Huan Wang, Min Zhao, Costan G. Magnussen, Bo Xi

**Affiliations:** ^1^ Department of Epidemiology/Shandong Provincial Clinical Research Center for Emergency and Critical Care Medicine, School of Public Health/Qilu Hospital, Cheeloo College of Medicine, Shandong University, Jinan, China; ^2^ Department of Toxicology and Nutrition, School of Public Health, Cheeloo College of Medicine, Shandong University, Jinan, China; ^3^ Menzies Institute for Medical Research, University of Tasmania, Hobart, TAS, Australia; ^4^ Research Centre of Applied and Preventive Cardiovascular Medicine, University of Turku, Turku, Finland; ^5^ Centre for Population Health Research, University of Turku and Turku University Hospital, Turku, Finland

**Keywords:** waist-to-height ratio, body mass index, waist circumference, left ventricular hypertrophy, geometric remodeling, children

## Abstract

**Background:**

Previous studies have shown that waist-to-height ratio (WHtR) performed similarly well when compared to body mass index (BMI) and waist circumference (WC) for identifying cardiovascular risk factors. However, to our knowledge, the performance of these three adiposity indices for identifying left ventricular hypertrophy (LVH) and left ventricular geometric (LVG) remodeling in youth has not been assessed. We aimed to determine the utility of BMI, WC and WHtR for identifying LVH and LVG in Chinese children.

**Methods:**

This study included 1,492 Chinese children aged 6-11 years. Adiposity indices assessed were BMI, WC and WHtR. LVH and high relative wall thickness (RWT) were defined using sex- and age-specific 90th percentile values of left ventricular mass index and RWT, respectively, based on the current population. LVG remodeling included concentric remodeling (CR), eccentric hypertrophy (EH) and concentric hypertrophy (CH), which was defined based on the combination of LVH and high RWT.

**Results:**

The magnitude of association of central obesity defined by WHtR with LVH [odds ratio (*OR*) =10.09, 95% confidence interval (*CI*) =6.66-15.29] was similar with general obesity defined by BMI (*OR*=10.49, 95% *CI*=6.97-15.80), and both were higher than central obesity defined by WC (*OR*=6.87, 95% *CI*=4.57-10.33). Compared with BMI, WHtR had better or similar predictive utility for identifying LVH, EH, and CH [the area under the curve (AUC): 0.84 *vs.* 0.79; 0.84 *vs.* 0.77; 0.87 *vs.* 0.88, respectively]; WC had worse or similar discriminatory utility with AUCs of 0.73, 0.70, 0.83, respectively.

**Conclusion:**

WHtR performed similarly or better than BMI or WC for identifying LVH and LVG remodeling among Chinese children. WHtR provides a simple and convenient measure of central obesity that might improve the discrimination of children with cardiac structural damage.

## Introduction

The prevalence of pediatric obesity has greatly increased worldwide, particularly in low- and middle-income countries (1, 2). In China, the prevalence of general obesity (3) and abdominal obesity (4) among children and adolescents has markedly increased over the past three decades. Obesity is related to cardiovascular (CV) risk (including hyperglycemia, elevated blood pressure, dyslipidemia, metabolic syndrome, and insulin resistance) (5) and short-term target organ damage (6, 7) in childhood. For example, obesity increases the risk of left ventricular hypertrophy (LVH) and left ventricular geometric (LVG) remodeling (8–11) (markers of cardiac structural damage), which are independent predictors of cardiovascular disease (CVD) (12, 13).

LVH was independently associated with the long term adverse CV events, such as coronary heart disease, other CVD related death, and heart failure (14). Specific abnormal LVG remodeling also provided distinct prognostic information. For example, hypertensive patients with concentric hypertrophy (CH) had the highest CV events and all-cause mortality, followed by those with eccentric hypertrophy (EH) and concentric remodeling (CR), compared with those with normal geometry (15). Importantly, participants converting from CR to normal geometry had decreased risk of all-cause mortality (16). LVH has been the most common target organ damage in children and adolescents with hypertension (17), and obesity is strongly associated with abnormal LVG modeling (8–11). Therefore, assessing the presence of LVH and LVG in the early life using a simple and effective obesity-related indicator could be helpful to prevent target organ damage such as LVG remodeling in the short term and the CVD outcomes in the long term.

Although BMI is universally used to determine obesity-related comorbidity and mortality, as an index of obesity it has several limitations. For example, sex- and age-specific cut-offs (18, 19) complicate the use of BMI to define obesity in practice, and BMI does not accurately discriminate body fat distribution (20). In contrast, WC and WHtR are markers of abdominal adiposity that are more closely linked with metabolic disturbances (21), and more strongly associated with CVD outcomes (22) and all-cause mortality (23). Sex- and age-specific cut-offs of WC are also required to define central obesity, whereas WHtR is standardized for height and indirectly adjusts for the effect of age, which is a simple and pragmatic index to correctly assess central obesity (24).

Although recent studies have reported that BMI, WC and WHtR performed similarly to predict common CV risk factors in children and adolescents (25–30), how these adiposity indices comparison when identifying LVH and LVG remodeling in children is largely unknown. A simplified and effective method to identify preclinical cardiac remodeling in the pediatric population is important. Therefore, we aimed to assess the utility of BMI, WC and WHtR for identifying LVH and LVG remodeling (including CR, EH and CH) and to determine the optimal cut-off of WHtR in Chinese children aged 6-11 years.

## Methods

### Subjects

This cross-sectional study included 1,492 children aged 6-11 years. Participants were recruited from one primary school in Huantai County, Zibo City, Shandong Province, China, using a convenient clustering sampling method from November 2017 to January 2018. Detailed information has been described elsewhere (31). Included participants provided written informed consent to participate in this study after knowing the aim and procedures. All children underwent physical measurements [height, weight, WC, and blood pressure (BP)] and an echocardiography examination at the school. A structured questionnaire was filled out by the children and their parents/guardians jointly at home. The study was approved by the Ethics Committees of the School of Public Health, Shandong University (Approval number: 20160308).

### Measurements

Height, weight and WC were measured twice in accordance with a uniform procedure and repeated a third time if the first two values differed by more than 1.0 cm (height), 0.5 kg (weight) or 1.0 cm (WC), with mean of the multiple measurements used for data analysis. A calibrated electronic weighing scale with automatic stadiometer (HGM-300; China) was used to measure height and weight, with children required to stand erect in bare feet and in light clothes. A non-elastic plastic tape was used to measure WC in a horizontal plane directly on the skin above 1 cm of the umbilicus after a normal exhalation. BMI was calculated as weight (kg) divided by the square of height (m^2^). WHtR was calculated as the ratio of WC (cm)/height (cm). The clinically validated and calibrated upper-arm electronic sphygmomanometer (Omron HEM-7012; Japan) was used to measure the BP in seated position, which is accurate at measuring BP among children aged under 18 years (32). Three BP readings were measured consecutively at one visit, with replicate readings performed after approximately 20-seconds, by trained staff following recommendations proposed by the Chinese working group of blood pressure measurement (33). Mean values of the last two BP readings were used for data analysis. Given the effect of children’s growth, the Z-scores for BMI, WC, WHtR and BP (the original values minus means specific for sex and age then divided by the standard deviations specific for sex and age) were calculated to reflect the sex- and age-specific distribution for these indices in this population and could be used for direct comparisons between different samples (e.g., with different ages and sexes).

A portable color Doppler Ultrasonography (CX30; USA) with 2-4 MHz convex array transducers was used to assess left ventricle structure. One experienced sonographer acquired all images following recommendations for cardiac chamber quantification (34). Left ventricular internal dimension (LVID), interventricular septal thickness (IVST) and left ventricular posterior wall thickness (LVPWT) were measured during diastole. In this study, the intra-class correlation coefficients for repeated measurements on the same 20 participants by the one operator were 0.92 for IVST and 0.95 for LVPWT. Left ventricular mass (LVM, g) was calculated as 0.8×1.04×[(LVID + IVST + LVPWT)^3^-(LVID)^3^] + 0.6 according to the Devereux’s formula (35). Left ventricular mass index (LVMI) was calculated as LVM (g) divided by height to the power of 2.7 (m^2.7^) (36). Relative wall thickness (RWT) was calculated as (LVPWT + IVST)/LVID (37).

A self-reported structured questionnaire was administered to collect information on lifestyle variables, including daily sleep duration, daily screen time, daily physical activity time, frequency of daily vegetable/fruit intake and weekly frequency of soft drink intake. Short sleep duration (< 9 hours per day) (38), long screen time (> 2 hours per day) (39), insufficient physical activity (< 1 hour per day) (40) and insufficient intake of vegetable/fruit (< 5 servings per day) (41) were defined according to pediatric recommendations. Finally, more frequent intake of soft drink (≥ 1 time per week) was defined based the distribution of frequency for our study population. The frequency of soft drink intake in the past 30 days was assessed using a self-designed questionnaire with 6 options: ‘never’, ‘less than once per month’, ‘1-3 times per month’, ‘1-2 times per week’, ‘3-5 times per week’, and ‘nearly everyday’. According to the distribution of frequency among this study population, we defined the more frequent intake of soft drink as more than once per week.

### Definitions of Obesity, LVH and LVG Remodeling

BMI-obese and WC-obese were defined using sex- and age-specific cut-offs of BMI and WC for Chinese children (19, 42, 43); WHtR-obese was defined as WHtR ≥ 0.5 (44). LVH was defined as LVMI ≥ sex- and age-specific 90th percentile and high RWT was defined as RWT ≥ sex- and age-specific 90th percentile for this population. LVG patterns were further categorized as: normal geometry (normal LVMI and normal RWT); CR (normal LVMI and high RWT); EH (LVH and normal RWT); and CH (LVH and high RWT) (45). In sensitivity analysis, sex- and age-specific 95th percentile values of LVMI and RWT for this population were used to re-define LVH, CR, EH and CH.

### Statistical Analysis

Continuous variables are expressed as means (standard deviations) and categorical variables as numbers (%); group differences are examined by the student’s t-test or chi-square test as appropriate. The proportions of LVH and LVG remodeling across obesity status subgroups defined by BMI, WC and WHtR were compared using the chi-square test. Multivariable logistic regression models were used to examine the association of obesity with LVH and LVG remodeling; adjusted odds ratios (*ORs*) and 95% confidence intervals (95% *CIs*) were estimated after adjusting for sex, age, daily sleep duration, daily screen time, daily physical activity time, daily frequency of vegetable/fruit intake, weekly frequency of soft drink intake, Z-scores for systolic and diastolic BP. The receiver operating characteristic (ROC) curve analysis was used to compare the performance of BMI, WC and WHtR for identifying LVH and LVG remodeling, with BMI as the referent, and the area under the ROC curve (AUC), sensitivity, specificity, positive predictive value (PPV) and negative predictive value (NPV) were calculated. Generally, an AUC value < 0.7 is considered poor, 0.7-0.8 as acceptable and > 0.8 as good (46). The optimal cut-off of WHtR in the present population was determined by maximizing the Youden index (sensitivity + specificity - 1) (47). All statistical analyses were conducted using SAS version 9.4 and R version 4.0.2 with “pROC” package (48); a two-sided *P* value < 0.05 was considered statistically significant.

## Results

### Participant Characteristics

A total of 1,492 children (boys: 53.2%) aged 6-11 years were included in this study. Among them, 146 children had LVH. Compared to children without LVH, those with LVH had higher levels, on average, of BMI, WC, WHtR, BMI Z-score, WC Z-score, WHtR Z-score, systolic and diastolic BP, and Z-scores for systolic and diastolic BP (all *P <*0.05, **Table 1**). Besides, sex- and age-adjusted means for BMI, WC, WHtR were still higher among children with LVH than those without LVH (BMI: 22.44 *vs.* 17.74 kg/m ^2^; WC: 72.88 *vs.* 61.91 cm; WHtR: 0.53 *vs.* 0.45). About two thirds of children with LVH were classified as obese, irrespective of the indices (BMI, WC or WHtR) used to define obesity, which was around three times as high as the proportion for those without LVH. A larger proportion of children without LVH (16.8%) had a short sleep duration (<9 hours/day) than those with LVH (8.9%); 8.2% of children with LVH had an exceeded screen time (>2 hours/day), nearly twice as high as those without LVH (4.3%) (**Table 1**). Children with CH had the highest levels of BMI, WC, WHtR, BMI Z-score, WC Z-score, WHtR Z-score and systolic BP, and those with normal geometry had the lowest levels. The proportions of obesity in children with abnormal geometry (CR: 31.2-42.2%; EH: 60.0-65.0%; CH: 80.4-87.0%) were much higher than those with normal geometry (15.8-25.8%) (**Supplementary Table 1** **)**.

**Table 1:** Characteristics of participants according to the presence of left ventricular hypertrophy.

Characteristics	Total (n = 1492)	LVH (n = 146)	Normal (n = 1346)	*P* value^*^
Age, years	8.90 (1.51)	8.87 (1.55)	8.91 (1.51)	0.750
Boys	793 (53.2)	78 (53.4)	715 (53.1)	0.944
BMI, kg/m ^2^	18.20 (3.45)	22.42 (4.46)	17.74 (2.99)	<0.001
WC, cm	62.98 (9.80)	72.80 (13.19)	61.92 (8.73)	<0.001
WHtR	0.46 (0.06)	0.53 (0.06)	0.45 (0.05)	<0.001
BMI Z-score	0.00 (1.00)	1.24 (1.17)	-0.14 (0.88)	<0.001
WC Z-score	0.00 (1.00)	1.05 (1.18)	-0.11 (0.90)	<0.001
WHtR Z-score	0.00 (1.00)	1.28 (1.04)	-0.14 (0.89)	<0.001
SBP, mmHg	106.34 (9.20)	108.63 (9.79)	106.09 (9.10)	0.002
DBP, mmHg	63.62 (6.68)	65.29 (7.58)	63.44 (6.55)	0.005
SBP Z-score	0.00 (1.00)	0.26 (0.98)	-0.03 (1.00)	0.001
DBP Z-score	0.00 (1.00)	0.26 (1.08)	-0.03 (0.98)	0.001
BMI-obese	327 (21.9)	97 (66.4)	230 (17.1)	<0.001
WC-obese	470 (31.5)	105 (71.9)	365 (27.1)	<0.001
WHtR-obese	363 (24.3)	99 (67.8)	264 (19.6)	<0.001
Sleep duration <9 hours/day	239 (16.0)	13 (8.9)	226 (16.8)	0.014
Screen time > 2 hours/day	70 (4.7)	12 (8.2)	58 (4.3)	0.034
Physical activity time < 1 hour/day	859 (57.6)	88 (60.3)	771 (57.3)	0.487
Intake of vegetable/fruit < 5 servings/day	1214 (81.4)	126 (86.3)	1088 (80.8)	0.107
Intake of soft drink ≥ 1 time/week	91 (6.1)	9 (6.2)	82 (6.1)	0.972

^*^Differences in characteristics between children with and without LVH were assessed using t test or chi-square test.

Continuous variables are expressed as means (standard deviations) and categorical variables as numbers (%).

LVH, left ventricular hypertrophy; BMI, body mass index; WC, waist circumference; WHtR, waist-to-height ratio; SBP, systolic blood pressure; DBP, diastolic blood pressure.

### Association of Indices of Obesity With LVH and LVG Remodeling

The prevalence of LVH, CR, EH and CH was higher in children with obesity, regardless of the measures (BMI, WC or WHtR) used to define obesity. Among those without obesity, CR was the prominent phenotype of LVG remodeling, while EH was the prominent phenotype in those with obesity (**Table 2** and **Supplementary Table 2**). The magnitude of the association of WHtR-obese with LVH (*OR* =10.09, 95% *CI* =6.66-15.29) was similar to the association observed for BMI-obese (*OR*=10.49, 95% *CI*=6.97-15.80), with both stronger than WC-obese (*OR*=6.87, 95% *CI*=4.57-10.33). WHtR-obese and BMI-obese also had a similar magnitude of association with LVG remodeling, which were both stronger than WC-obese. Besides, the continuous Z-scores for BMI, WC and WHtR were also positively associated with LVH and LVG remodeling (**Table 3**). Sensitivity analysis showed similar associations of obesity with LVH and LVG remodeling (**Supplementary Table 3**).

**Table 2:** Prevalence of left ventricular hypertrophy and left ventricular geometric remodeling according to obesity status, n (%).

Obesity status	LVH	LVG remodeling		
		CR	EH	CH
**BMI**				
Normal (n=1165)	49 (4.2)	75 (6.4)	40 (3.4)	9 (0.8)
Obese (n=327)	97 (29.7)	34 (10.4)	60 (18.4)	37 (11.3)
* P* value^*^	<0.001	0.015	<0.001	<0.001
**WC**				
Normal (n=1022)	41 (4.0)	63 (6.2)	35 (3.4)	6 (0.6)
Obese (n=470)	105 (22.3)	46 (9.8)	65 (13.8)	40 (8.5)
* P* value^*^	<0.001	0.013	<0.001	<0.001
**WHtR**				
Normal (n=1129)	47 (4.2)	70 (6.2)	38 (3.4)	9 (0.8)
Obese (n=363)	99 (27.3)	39 (10.7)	62 (17.1)	37 (10.2)
* P* value^*^	<0.001	0.004	<0.001	<0.001

^*^ Differences in the prevalence of LVH or LVG between non-obese and obese groups were assessed using chi-square test.

LVH, left ventricular hypertrophy; LVG, left ventricular geometric; CR, concentric remodeling; EH, eccentric hypertrophy; CH, concentric hypertrophy; BMI, body mass index; WC, waist circumference; WHtR, waist-to-height ratio.

**Table 3:** Association of obesity with left ventricular hypertrophy and left ventricular geometric remodeling.

	Model 1		Model 2		Model 3	
	*OR* (95% *CI*)	*P* value	*OR* (95% *CI*)	*P* value	*OR* (95% *CI*)	*P* value
**LVH**						
BMI-obese	9.73 (6.70-14.14)	<0.001	9.76 (6.70-14.23)	<0.001	10.49 (6.97-15.80)	<0.001
WC-obese	6.92 (4.73-10.13)	<0.001	6.75 (4.60-9.90)	<0.001	6.87 (4.57-10.33)	<0.001
WHtR-obese	9.58 (6.52-14.08)	<0.001	9.75 (6.60-14.39)	<0.001	10.09 (6.66-15.29)	<0.001
BMI Z-score	3.38 (2.82-4.07)	<0.001	3.41 (2.83-4.11)	<0.001	3.96 (3.20-4.91)	<0.001
WC Z-score	2.80 (2.36-3.32)	<0.001	2.81 (2.36-3.34)	<0.001	3.15 (2.58-3.85)	<0.001
WHtR Z-score	3.69 (3.04-4.48)	<0.001	3.76 (3.08-4.58)	<0.001	4.30 (3.44-5.38)	<0.001
**CR**						
BMI-obese	2.42 (1.57-3.74)	<0.001	2.49 (1.61-3.86)	<0.001	2.16 (1.36-3.44)	0.001
WC-obese	2.11 (1.41-3.14)	<0.001	2.21 (1.47-3.31)	<0.001	1.95 (1.26-3.00)	0.003
WHtR-obese	2.62 (1.71-4.02)	<0.001	2.74 (1.78-4.22)	<0.001	2.45 (1.56-3.86)	<0.001
BMI Z-score	1.64 (1.34-2.00)	<0.001	1.67 (1.37-2.05)	<0.001	1.59 (1.28-1.99)	<0.001
WC Z-score	1.74 (1.44-2.10)	<0.001	1.78 (1.47-2.16)	<0.001	1.73 (1.39-2.15)	<0.001
WHtR Z-score	1.54 (1.26-1.89)	<0.001	1.58 (1.28-1.94)	<0.001	1.49 (1.19-1.86)	<0.001
**EH**						
BMI-obese	8.11 (5.27-12.46)	<0.001	8.21 (5.32-12.68)	<0.001	8.18 (5.12-13.09)	<0.001
WC-obese	5.40 (3.51-8.31)	<0.001	5.26 (3.41-8.11)	<0.001	4.97 (3.13-7.91)	<0.001
WHtR-obese	8.27 (5.31-12.89)	<0.001	8.51 (5.44-13.33)	<0.001	8.28 (5.14-13.35)	<0.001
BMI Z-score	3.26 (2.66-4.01)	<0.001	3.30 (2.68-4.07)	<0.001	3.69 (2.90-4.68)	<0.001
WC Z-score	2.75 (2.26-3.35)	<0.001	2.77 (2.27-3.39)	<0.001	2.99 (2.37-3.77)	<0.001
WHtR Z-score	3.67 (2.95-4.55)	<0.001	3.75 (3.01-4.69)	<0.001	4.17 (3.25-5.35)	<0.001
**CH**						
BMI-obese	21.94 (10.40-46.28)	<0.001	21.74 (10.26-46.07)	<0.001	26.02 (11.72-57.76)	<0.001
WC-obese	19.06 (8.00-45.39)	<0.001	19.03 (7.95-45.55)	<0.001	21.44 (8.69-52.87)	<0.001
WHtR-obese	20.24 (9.48-43.21)	<0.001	20.56 (9.56-44.23)	<0.001	23.17 (10.38-51.73)	<0.001
BMI Z-score	4.63 (3.44-6.21)	<0.001	4.64 (3.43-6.28)	<0.001	5.81 (4.11-8.21)	<0.001
WC Z-score	3.73 (2.80-4.98)	<0.001	3.72 (2.77-4.99)	<0.001	4.48 (3.20-6.28)	<0.001
WHtR Z-score	4.50 (3.32-6.08)	<0.001	4.56 (3.33-6.23)	<0.001	5.47 (3.84-7.78)	<0.001

OR, odds ratio; CI, confidence interval; LVH, left ventricular hypertrophy; CR, concentric remodeling; EH, eccentric hypertrophy; CH, concentric hypertrophy; BMI, body mass index; WC, waist circumference; WHtR, waist-to-height ratio.

Model 1: Adjusted for sex and age.

Model 2: Model 1 + daily sleep duration, daily screen time, daily physical activity, frequency of daily vegetable/fruit intake and frequency of weekly soft drink intake.

Model 3: Model 2 + Z-scores for systolic and diastolic blood pressure.

### Utility of Adiposity Indices for Identifying LVH and LVG Remodeling

**Figure 1:**
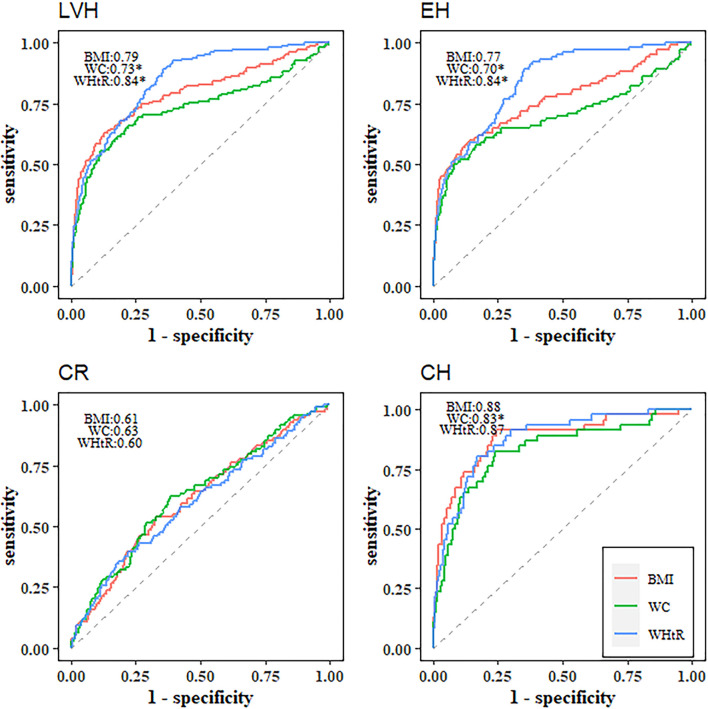
Receiver operating characteristic curves of BMI, WC and WHtR for identifying left ventricular hypertrophy and left ventricular geometric remodeling. ^*^Statistically significant difference in area under the operating characteristic curves as compared with BMI. LVH, left ventricular hypertrophy; CR, concentric remodeling; EH, eccentric hypertrophy; CH, concentric hypertrophy; BMI, body mass index; WC, waist circumference; WHtR, waist-to-height ratio.

**Figure 2:**
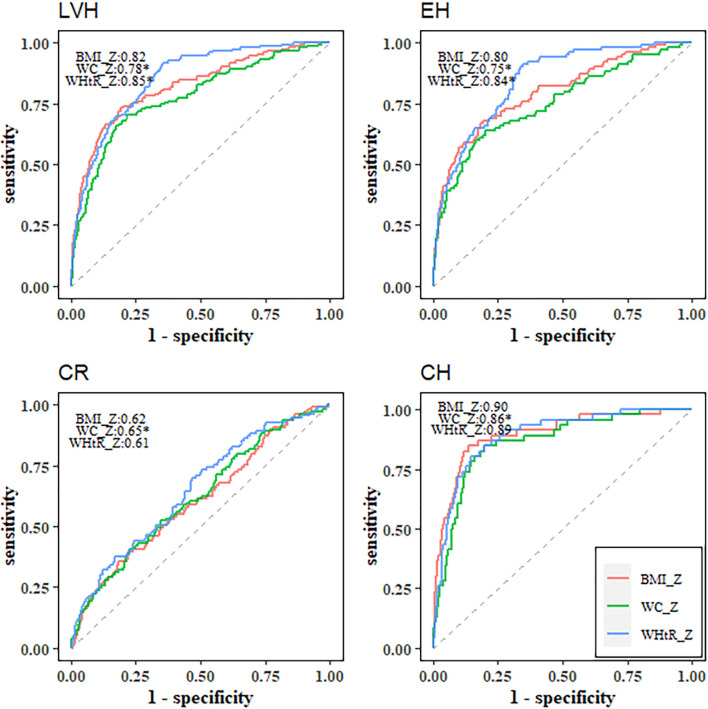
Receiver operating characteristic curves of BMI Z-score, WC Z-score and WHtR Z-score for identifying left ventricular hypertrophy and left ventricular geometric remodeling. ^*^Statistically significant difference in area under the operating characteristic curves as compared with BMI Z-score. LVH, left ventricular hypertrophy; CR, concentric remodeling; EH, eccentric hypertrophy; CH, concentric hypertrophy; BMI, body mass index; WC, waist circumference; WHtR, waist-to-height ratio.

Compared with BMI (AUC=0.79, 95% *CI*: 0.75-0.84), WC (AUC=0.73, 95% *CI*: 0.68-0.79) had worse predictive utility for identifying LVH, while WHtR (AUC=0.84, 95% *CI*: 0.81-0.88) outperformed BMI (**Table 4** and **Figure 1**). BMI, WC and WHtR had similarly poor discriminatory utility for CR (all AUCs below 0.7) (**Table 4** and **Figure 1**). For identifying EH, WC (AUC=0.70, 95% *CI*: 0.63-0.77) was inferior to BMI (AUC=0.77, 95% *CI*: 0.71-0.82), and WHtR (AUC=0.84, 95% *CI*: 0.80-0.88) had the best performance (**Table 4** and **Figure 1**). For identifying CH, WC (AUC=0.83, 95% *CI*: 0.76-0.90) had a worse utility in comparison to BMI (AUC=0.88, 95% *CI*: 0.82-0.94) and WHtR (AUC=0.87, 95% *CI*: 0.82-0.92) (**Table 4** and **Figure 1**). Z-scores of these three adiposity indices showed largely similar discriminatory utility as those using the original scales (**Table 4** and **Figure 2**). Similar results were observed in the sensitivity analysis (**Supplementary Table 4**).

**Table 4:** Utility of adiposity indices for identifying left ventricular hypertrophy and left ventricular geometric remodeling.

	AUC (95% *CI*)	*P* value^*^	Sensitivity, %	Specificity, %	PPV, %	NPV, %
**LVH**						
BMI	0.79 (0.75-0.84)	Ref.	63.0	87.2	34.7	95.6
WC	0.73 (0.68-0.79)	<0.001	55.5	88.6	34.6	94.8
WHtR	0.84 (0.81-0.88)	0.001	89.7	63.7	21.2	98.3
BMI Z-score	0.82 (0.78-0.86)	Ref.	73.3	80.8	29.2	96.5
WC Z-score	0.78 (0.73-0.82)	<0.001	70.6	78.5	26.2	96.1
WHtR Z-score	0.85 (0.82-0.88)	0.007	91.1	65.1	22.1	98.5
**CR**						
BMI	0.61 (0.56-0.67)	Ref.	54.1	67.4	12.8	94.3
WC	0.63 (0.57-0.68)	0.193	62.4	61.8	12.6	94.9
WHtR	0.60 (0.54-0.66)	0.493	34.9	82.8	15.1	93.5
BMI Z-score	0.62 (0.56-0.67)	Ref.	52.3	65.6	11.8	94.0
WC Z-score	0.65 (0.59-0.70)	0.011	73.4	49.5	11.4	95.5
WHtR Z-score	0.61 (0.55-0.66)	0.282	35.8	81.9	14.8	93.5
**EH**						
BMI	0.77 (0.71-0.82)	Ref.	60.0	86.0	25.8	96.4
WC	0.70 (0.63-0.77)	<0.001	50.0	92.0	33.6	95.8
WHtR	0.84 (0.80-0.88)	<0.001	92.0	61.9	16.3	99.0
BMI Z-score	0.80 (0.75-0.85)	Ref.	67.0	81.8	23.0	96.8
WC Z-score	0.75 (0.70-0.81)	0.001	64.0	79.9	20.5	96.5
WHtR Z-score	0.84 (0.81-0.88)	0.001	91.0	65.4	17.5	98.9
**CH**						
BMI	0.88 (0.82-0.94)	Ref.	91.3	75.7	12.2	99.6
WC	0.83 (0.76-0.90)	<0.001	82.6	76.2	11.4	99.2
WHtR	0.87 (0.82-0.92)	0.866	80.4	83.4	15.2	99.1
BMI Z-score	0.90 (0.85-0.95)	Ref.	84.8	86.5	18.9	99.4
WC Z-score	0.86 (0.81-0.92)	0.033	84.8	80.1	13.7	99.3
WHtR Z-score	0.89 (0.85-0.94)	0.741	80.4	85.6	17.2	99.2

LVH, left ventricular hypertrophy; CR, concentric remodeling; EH, eccentric hypertrophy; CH, concentric hypertrophy; BMI, body mass index; WC, waist circumference; WHtR, waist-to-height ratio; AUC, area under the operating characteristic curve; CI, confidence interval; PPV, positive predictive value; NPV, negative predictive value; Ref, referent.

^*^Comparisons of AUCs with BMI as the referent.

**Figure 3:**
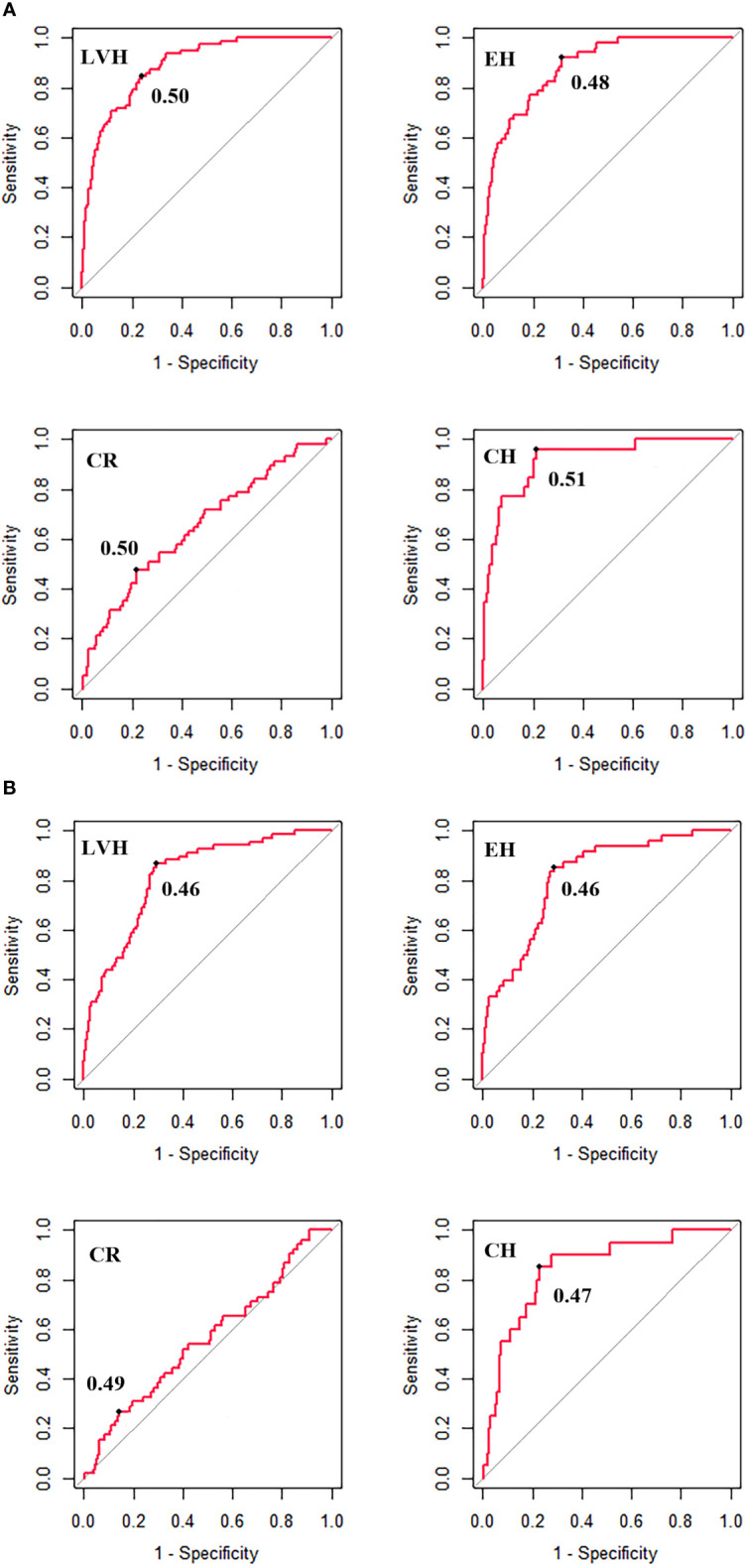
The optimal cut-off of WHtR for identifying left ventricular hypertrophy and left ventricular geometric remodeling for boys **(A)** and girls **(B)**. LVH, left ventricular hypertrophy; CR, concentric remodeling; EH, eccentric hypertrophy; CH, concentric hypertrophy; BMI, body mass index; WC, waist circumference; WHtR, waist-to-height ratio. The optimal cut-off is the threshold corresponding to the maximum of Youden Index (sensitivity + specificity -1).

In addition, the optimal cut-offs of WHtR to identify LVH, CR, EH and CH were around 0.50 for boys (0.50, 0.50, 0.48, 0.51, respectively), while the corresponding optimal cut-offs were somewhat lower than 0.50 for girls (0.46, 0.49, 0.46, 0.47, respectively) (**Figure 3**). Except for CR, the sensitivity of WHtR was above 0.80, suggesting that WHtR could perform well to correctly identify children with LVH, EH and CH (**Table 4**).

## Discussion

In this population-based study of Chinese children aged 6-11 years, we found that the magnitude of association of central obesity defined by WHtR with LVH and LVG remodeling was similar with obesity defined by BMI, and both were stronger than central obesity defined by WC; WHtR performed similarly or better than BMI or WC for discriminating LVH and LVG remodeling; and WHtR cut-offs of ~0.5 for boys and <0.5 for girls provided the best discrimination of those with LVH and LVG remodeling. Our findings contribute to the evidence base that support the use of WHtR to identify children at risk of cardio-metabolic outcomes by extending them to include subclinical cardiac structural damage.

Consistent with previous studies (10, 49–51), we found obesity was associated with LVH, and the magnitude of the association was stronger for central obesity defined by WHtR and general obesity defined by BMI than central obesity defined by WC. A cross-sectional study including 281 outpatient children aged 6-16 years found that WHtR was significantly associated with LVH, while BMI or WC was not (51). However, another cross-sectional study among 96 children and adolescents aged 7-15 years showed that WHtR was less strongly correlated with LVMI among these three adiposity indices (52). Differences in demographic characteristics (e.g., different distributions of sex and age) and various methods for measurements of anthropometric and cardiac structural indices (e.g., different positions and devices during measurements) might explain these inconsistent results.

In our study, obesity was also associated with LVG remodeling, and the magnitude of the association of obesity with CH was the strongest, followed by EH and CR. A study including 62 normotensive children aged 8-11 years showed that elevated WHtR increased the odds of any phenotype of LVG remodeling (8). A study among 526 children aged 6-15 years found that neither obesity (defined by BMI) nor WC Z-score was associated with CR, whereas obesity and WC Z-score was independently associated with EH and CH (11). Another study including 343 African American youths with a mean age of 13.8 years reported that obesity (defined by BMI) was associated with CR independent of BP, but not with CH (53). The heterogeneity of ethnicity, the definition of LVG remodeling patterns and the consideration of potential covariates might partially explain these inconsistent findings.

Previous studies have compared the predictive utility of BMI, WC and WHtR for common CV risk factors in children and adolescents (25–30, 54–56) with most reporting similar performance among these three adiposity indices (25–30). However, few studies have assessed the performance for identifying LVH and LVG remodeling among children. A cross-sectional study of 10,907 adults from China reported no discrimination utility of BMI, WC or WHtR for CR (all AUCs <0.5), and similarly fair performance for EH and CH (AUCs ranging from 0.63 to 0.72) (57). Another study including 281 white children aged 6-16 years reported that WHtR performed best for identifying LVH compared with BMI or WC, with AUCs (95% *CI*) of 0.711 (0.650-0.733), 0.680 (0.616-0.743) and 0.657 (0.593-0.722), respectively (51). Likewise, our study reported that WHtR performed similarly or better than BMI or WC for identifying LVH and LVG remodeling; suggesting the use of WHtR to screen CV risk factors in practice could improve the ability to identify youth at high risk of cardiac remodeling. It has been shown that WHtR could outperform BMI and WC to predict total and trunk adiposity in children and adolescents (58). And increased adiposity may contribute to cardiac remodeling by hemodynamic and metabolic pathways, including increase in stroke volume and cardiac output, disorder of cardiac metabolism, activation of sympathetic nervous system, and secretion of adipokines by the adipose tissue (10, 59).

However, we found that all three adiposity indices had low predictive utility of CR with the AUCs of around 0.6, suggesting that a single index alone might have limited value to predict this remodeling phenotype in children. In addition, generally low PPVs but high NPVs suggest the better utility for identifying those without abnormal cardiac structures, which might be explained by the low prevalence of LVH/LVG remodeling among children.

It has been generally accepted that a WHtR cut-off of 0.5 might be useful in predicting CV risk in youth, independent of sex, age or ethnicity (44, 60), whereas a study including children and adolescents aged 7-19 years from Europe (13,172; boys: 49.7%) and southern China (14,566; boys: 50.3%) reported that a WHtR cut-off of 0.5 was not suitable for diverse ethnic groups, with a lower threshold of WHtR proposed, especially for girls from southern China (61). Another multicenter study involving 8,130 children and adolescents aged 7-18 years (boys: 53.2%) from China suggested that the optimal WHtR cut-off of 0.467 would be more accurate to identify the clustering of CV risk factors in the pediatric population, and that the optimal cut-offs varied across sex and age with lower cut-offs for girls and those aged 12 years or older (62). In our study, the optimal cut-offs of WHtR were ~0.5 for boys and <0.5 for girls, which may support the sex dependent cut-offs of WHtR. Besides, there were some differences in basic characteristics between boys and girls in our study (**Supplementary Table 5**). For example, boys had higher BMI, WC, WHtR, and SBP than girls, and boys tended to have unhealthy eating habits (such as insufficient intake of vegetable/fruit and more frequent intake of soft drink) compared with girls. Sex hormones (e.g., estrogens and androgens) and genetic mechanisms could regulate glucose and lipid homeostasis, energy metabolism and gene expression in a sexually dimorphic manner resulting in sex-specific cardio-metabolic disorders (63). Therefore, sex dependent cut-offs for identifying LVH/LVG are necessary according to these different sex-specific features in cardio-metabolic disorders. Importantly, a simple and effective index for screening obesity in youth could be useful to identify obesity-related CV risk factors and to prevent the target organ damage in the early stage, and further cohort studies with a large sample size are needed to confirm our findings.

The strengths of our study were that we compared the discriminatory capability of BMI, WC and WHtR and determined the optimal cut-off of WHtR based on more clinically relevant markers of target organ damage, LVH and LVG remodeling, among children in a relatively large sample. However, our study has several limitations. First, the design of this study was cross-sectional and the interpretation of predictive utility of these three adiposity indices should be made with caution as we are unable to discount reverse causation. Second, the present study only included children aged 6-11 years from one primary school, which limits the generalizability of our findings. Third, despite a standard questionnaire being used to collect lifestyle variables, only diet frequency was assessed without more details about food quality and quantity, and puberty status was not evaluated in the present study. Therefore, it is possible that our estimates might be biased due to unmeasured or residual confounding.

In summary, WHtR performed similarly well or better than BMI or WC for identifying LVH and LVG remodeling, with a WHtR cut-off of ~0.5 for boys and <0.50 for girls shown to have the best discriminatory utility. Our data suggest that WHtR could be used to identify children at risk of having subclinical cardiac structural damage.

## Data Availability Statement

The original contributions presented in the study are included in the article/**Supplementary Material**. Further inquiries can be directed to the corresponding author.

## Ethics Statement

The studies involving human participants were reviewed and approved by Ethics Committees of the School of Public Health, Shandong University (Approval number: 20160308). Written informed consent to participate in this study was provided by the participants’ legal guardian/next of kin.

## Author Contributions

BX conceptualized and designed the study, supervised the data collation, statistical analyses, and reviewed and revised the manuscript. HW did the statistical analyses, drafted the initial manuscript, and reviewed and revised the manuscript. CM, MZ, and BX reviewed and revised the manuscript. All authors contributed to the article and approved the submitted version.

## Funding

This work was supported by the National Natural Science Foundation of China (81722039, 81673195).

## Conflict of Interest

The authors declare that the research was conducted in the absence of any commercial or financial relationships that could be construed as a potential conflict of interest.

## Publisher’s Note

All claims expressed in this article are solely those of the authors and do not necessarily represent those of their affiliated organizations, or those of the publisher, the editors and the reviewers. Any product that may be evaluated in this article, or claim that may be made by its manufacturer, is not guaranteed or endorsed by the publisher.
