# Supplementary material for: Utility of Three Adiposity Indices for Identifying Left Ventricular Hypertrophy and Geometric Remodeling in Chinese Children
Source: Front Endocrinol (Lausanne). 2021 Nov 16;12:762250. doi: 10.3389/fendo.2021.762250 (PMC8636598; doi:10.3389/fendo.2021.762250)
Supplement: Supplementary file 1 [file DataSheet_1.doc]

**Table S1. Characteristics of participants across the four patterns of left ventricular geometric remodeling (definitions based on 90th percentile values of this study population)**

| Characteristics | Total (n=1492) | LVG patterns | | | | *P* value^*^ |
| --- | --- | --- | --- | --- | --- | --- |
|  |  | NG (n=1237) | CR (n=109) | EH (n=100) | CH (n=46) |  |
| Age, years | 8.90 (1.51) | 8.91 (1.51) | 8.94 (1.50) | 8.76 (1.54) | 9.09 (1.57) | 0.670 |
| Boys | 793 (53.2) | 658 (53.2) | 57 (52.3) | 52 (52.0) | 26 (56.5) | 0.961 |
| BMI, kg/m ^2^ | 18.20 (3.45) | 17.63 (2.90) | 19.00 (3.68) | 22.00 (4.72) | 23.32 (3.71) | <0.001 |
| WC, cm | 62.98 (9.80) | 61.56 (8.49) | 65.94 (10.28) | 71.84 (14.15) | 74.87 (10.67) | <0.001 |
| WHtR | 0.46 (0.06) | 0.45 (0.05) | 0.47 (0.06) | 0.53 (0.06) | 0.54 (0.06) | <0.001 |
| BMI Z-score | 0.00 (1.00) | -0.17 (0.85) | 0.26 (1.08) | 1.10 (1.21) | 1.55 (1.03) | <0.001 |
| WC Z-score | 0.00 (1.00) | -0.16 (0.87) | 0.37 (1.07) | 0.94 (1.24) | 1.31 (1.00) | <0.001 |
| WHtR Z-score | 0.00 (1.00) | -0.17 (0.87) | 0.20 (1.00) | 1.20 (1.08) | 1.43 (0.94) | <0.001 |
| SBP, mmHg | 106.34 (9.20) | 105.89 (9.10) | 108.34 (8.88) | 108.58 (10.30) | 108.74 (8.67) | 0.001 |
| DBP, mmHg | 63.62 (6.68) | 63.34 (6.48) | 64.63 (7.21) | 65.65 (8.03) | 64.52 (6.49) | 0.002 |
| SBP Z-score | 0.00 (1.00) | -0.06 (1.00) | 0.24 (0.95) | 0.27 (1.02) | 0.25 (0.89) | <0.001 |
| DBP Z-score | 0.00 (1.00) | -0.04 (0.97) | 0.15 (1.06) | 0.31 (1.12) | 0.15 (0.99) | 0.001 |
| BMI-obese | 327 (21.9) | 196 (15.8) | 34 (31.2) | 60 (60.0) | 37 (80.4) | <0.001 |
| WC-obese | 470 (31.5) | 319 (25.8) | 46 (42.2) | 65 (65.0) | 40 (87.0) | <0.001 |
| WHtR-obese | 363 (24.3) | 225 (18.2) | 39 (35.8) | 62 (62.0) | 37 (80.4) | <0.001 |
| Sleep duration < 9 hours/day | 239 (16.0) | 211 (17.1) | 15 (13.8) | 8 (8.0) | 5 (10.9) | 0.069 |
| Screen time > 2 hours/day | 70 (4.7) | 54 (4.4) | 4 (3.7) | 6 (6.0) | 6 (13.0) | 0.044 |
| Physical activity time < 1 hour/day | 859 (57.6) | 715 (57.8) | 56 (51.4) | 60 (60.0) | 28 (60.9) | 0.535 |
| Intake of vegetable/fruit <5 servings/day | 1214 (81.4) | 1008 (81.5) | 80 (73.4) | 89 (89.0) | 37 (80.4) | 0.038 |
| Intake of soft drink ≥ 1 time/week | 91 (6.1) | 75 (6.1) | 7 (6.4) | 5 (5.0) | 4 (8.7) | 0.856 |

Continuous variables are expressed as means (standard deviations) and categorical variables as numbers (%).

LVG, left ventricular geometric; NG, normal geometry; CR, concentric remodeling; EH, eccentric hypertrophy; CH, concentric hypertrophy; BMI, body mass index; WC, waist circumference; WHtR, waist-to-height ratio; SBP, systolic blood pressure; DBP, diastolic blood pressure.

^*^Differences in characteristics across the four patterns of LVG were assessed using variance analysis or chi-square test as appropriate.

**Table S2. Prevalence of left ventricular hypertrophy and left ventricular geometric remodeling according to obesity status, n (%) (definitions based on 95th percentile values of this study population)**

| Obesity status | LVH | LVG remodeling | | |
| --- | --- | --- | --- | --- |
|  |  | CR | EH | CH |
| **BMI** |  |  |  |  |
| Normal (n=1165) | 21 (1.8) | 37 (3.2) | 19 (1.6) | 2 (0.2) |
| Obese (n=327) | 49 (15.0) | 26 (8.0) | 34 (10.4) | 15 (4.6) |
| *P* value^*^ | <0.001 | <0.001 | <0.001 | <0.001 |
| **WC** |  |  |  |  |
| Normal (n=1022) | 19 (1.9) | 28 (2.7) | 17 (1.7) | 2 (0.2) |
| Obese (n=470) | 51 (10.9) | 35 (7.5) | 36 (7.7) | 15 (3.2) |
| *P* value^*^ | <0.001 | <0.001 | <0.001 | <0.001 |
| **WHtR** |  |  |  |  |
| Normal (n=1129) | 21 (1.9) | 32 (2.8) | 18 (1.6) | 3 (0.3) |
| Obese (n=363) | 49 (13.5) | 31 (8.5) | 35 (9.6) | 14 (3.9) |
| *P* value^*^ | <0.001 | <0.001 | <0.001 | <0.001 |

**^*^**Differences in the prevalence of LVH or LVG between non-obese and obese groups were assessed using chi-square test.

LVH, left ventricular hypertrophy; LVG, left ventricular geometric; CR, concentric remodeling; EH, eccentric hypertrophy; CH, concentric hypertrophy; BMI, body mass index; WC, waist circumference; WHtR, waist-to-height ratio.

**Table S3. Association of obesity with left ventricular hypertrophy and left ventricular geometric remodeling (definitions based on 95th percentile values of this study population)**

|  | Model 1 | |  | Model 2 | |  | Model 3 | |
| --- | --- | --- | --- | --- | --- | --- | --- | --- |
|  | *OR* (95% *CI*) | *P* value |  | *OR* (95% *CI*) | *P* value |  | *OR* (95% *CI*) | *P* value |
| **LVH** |  |  |  |  |  |  |  |  |
| BMI-obese | 9.80 (5.77-16.64) | <0.001 |  | 9.57 (5.62-16.30) | <0.001 |  | 10.40 (5.86-18.46) | <0.001 |
| WC-obese | 6.49 (3.78-11.13) | <0.001 |  | 6.33 (3.68-10.90) | <0.001 |  | 6.43 (3.62-11.42) | <0.001 |
| WHtR-obese | 9.23 (5.38-15.83) | <0.001 |  | 9.12 (5.30-15.69) | <0.001 |  | 9.57 (5.37-17.07) | <0.001 |
| BMI Z-score | 3.25 (2.59-4.08) | <0.001 |  | 3.24 (2.58-4.08) | <0.001 |  | 3.81 (2.92-4.97) | <0.001 |
| WC Z-score | 2.75 (2.20-3.42) | <0.001 |  | 2.72 (2.18-3.41) | <0.001 |  | 3.09 (2.38-4.00) | <0.001 |
| WHtR Z-score | 3.30 (2.61-4.15) | <0.001 |  | 3.31 (2.61-4.20) | <0.001 |  | 3.81 (2.91-5.00) | <0.001 |
| **CR** |  |  |  |  |  |  |  |  |
| BMI-obese | 3.08 (1.83-5.19) | <0.001 |  | 3.21 (1.90-5.44) | <0.001 |  | 2.81 (1.60-4.92) | <0.001 |
| WC-obese | 3.17 (1.90-5.29) | <0.001 |  | 3.36 (2.00-5.64) | <0.001 |  | 3.02 (1.74-5.23) | <0.001 |
| WHtR-obese | 3.83 (2.27-6.47) | <0.001 |  | 4.11 (2.42-6.99) | <0.001 |  | 3.71 (2.13-6.48) | <0.001 |
| BMI Z-score | 2.05 (1.63-2.57) | <0.001 |  | 2.13 (1.68-2.69) | <0.001 |  | 2.12 (1.63-2.75) | <0.001 |
| WC Z-score | 2.03 (1.62-2.56) | <0.001 |  | 2.12 (1.68-2.69) | <0.001 |  | 2.13 (1.63-2.77) | <0.001 |
| WHtR Z-score | 1.87 (1.48-2.36) | <0.001 |  | 1.95 (1.53-2.48) | <0.001 |  | 1.88 (1.45-2.44) | <0.001 |
| **EH** |  |  |  |  |  |  |  |  |
| BMI-obese | 7.94 (4.44-14.17) | <0.001 |  | 7.82 (4.36-14.01) | <0.001 |  | 7.79 (4.14-14.65) | <0.001 |
| WC-obese | 5.43 (3.01-9.79) | <0.001 |  | 5.24 (2.90-9.49) | <0.001 |  | 4.94 (2.62-9.32) | <0.001 |
| WHtR-obese | 8.15 (4.48-14.82) | <0.001 |  | 8.11 (4.44-14.82) | <0.001 |  | 7.98 (4.19-15.18) | <0.001 |
| BMI Z-score | 3.40 (2.63-4.38) | <0.001 |  | 3.42 (2.64-4.43) | <0.001 |  | 3.93 (2.92-5.29) | <0.001 |
| WC Z-score | 2.79 (2.17-3.58) | <0.001 |  | 2.80 (2.17-3.61) | <0.001 |  | 3.07 (2.29-4.13) | <0.001 |
| WHtR Z-score | 3.41 (2.63-4.42) | <0.001 |  | 3.46 (2.65-4.52) | <0.001 |  | 3.88 (2.87-5.26) | <0.001 |
| **CH** |  |  |  |  |  |  |  |  |
| BMI-obese | 34.90 (7.90-154.16) | <0.001 |  | 33.72 (7.59-149.90) | <0.001 |  | 45.75 (9.70-215.84) | <0.001 |
| WC-obese | 19.37 (4.40-85.22) | <0.001 |  | 20.26 (4.55-90.12) | <0.001 |  | 24.63 (5.31-114.20) | <0.001 |
| WHtR-obese | 21.62 (6.05-77.25) | <0.001 |  | 21.70 (6.00-78.56) | <0.001 |  | 26.63 (6.96-101.80) | <0.001 |
| BMI Z-score | 3.88 (2.53-5.93) | <0.001 |  | 3.83 (2.48-5.92) | <0.001 |  | 4.83 (2.96-7.90) | <0.001 |
| WC Z-score | 3.42 (2.21-5.29) | <0.001 |  | 3.34 (2.15-5.19) | <0.001 |  | 4.15 (2.51-6.87) | <0.001 |
| WHtR Z-score | 3.75 (2.43-5.78) | <0.001 |  | 3.73 (2.38-5.85) | <0.001 |  | 4.59 (2.76-7.63) | <0.001 |

*OR*, odds ratio; *CI*, confidence interval; LVH, left ventricular hypertrophy; CR, concentric remodeling; EH, eccentric hypertrophy; CH, concentric hypertrophy; BMI, body mass index; WC, waist circumference; WHtR, waist-to-height ratio.

Model 1: Adjusted for sex and age.

Model 2: Model 1 + daily sleep duration, daily screen time, daily physical activity, frequency of daily vegetable/fruit intake and frequency of weekly soft drink intake.

Model 3: Model 2 + Z-scores for systolic and diastolic blood pressure.

**Table S4. Utility of adiposity indices in identifying left ventricular hypertrophy and left ventricular geometric remodeling (definitions based on 95th percentile values of this study population)**

|  | AUC (95% *CI*) | *P* value^*^ | Sensitivity, % | Specificity, % | PPV, % | NPV, % |
| --- | --- | --- | --- | --- | --- | --- |
| **LVH** |  |  |  |  |  |  |
| BMI | 0.81 (0.75-0.87) | Ref. | 67.1 | 83.5 | 16.7 | 98.1 |
| WC | 0.73 (0.65-0.81) | <0.001 | 60.0 | 86.5 | 18.0 | 97.8 |
| WHtR | 0.84 (0.79-0.88) | 0.110 | 92.9 | 60.3 | 10.3 | 99.4 |
| BMI Z-score | 0.84 (0.79-0.89) | Ref. | 78.6 | 78.1 | 15.0 | 98.7 |
| WC Z-score | 0.78 (0.72-0.84) | 0.001 | 67.1 | 81.7 | 15.3 | 98.1 |
| WHtR Z-score | 0.85 (0.81-0.89) | 0.452 | 75.7 | 81.0 | 16.4 | 98.6 |
| **CR** |  |  |  |  |  |  |
| BMI | 0.68 (0.61-0.75) | Ref. | 65.1 | 65.0 | 7.9 | 97.6 |
| WC | 0.68 (0.61-0.75) | 0.999 | 63.5 | 68.7 | 8.6 | 97.6 |
| WHtR | 0.67 (0.60-0.74) | 0.704 | 49.2 | 79.2 | 9.9 | 97.1 |
| BMI Z-score | 0.70 (0.63-0.76) | Ref. | 68.3 | 63.4 | 8.0 | 97.7 |
| WC Z-score | 0.72 (0.65-0.78) | 0.184 | 82.5 | 51.7 | 7.3 | 98.5 |
| WHtR Z-score | 0.68 (0.61-0.75) | 0.224 | 55.6 | 73.4 | 8.8 | 97.3 |
| **EH** |  |  |  |  |  |  |
| BMI | 0.80 (0.73-0.86) | Ref. | 62.3 | 84.4 | 13.5 | 98.3 |
| WC | 0.71 (0.62-0.80) | <0.001 | 58.5 | 84.3 | 12.7 | 98.1 |
| WHtR | 0.84 (0.79-0.89) | 0.071 | 92.5 | 62.0 | 8.7 | 99.5 |
| BMI Z-score | 0.84 (0.79-0.90) | Ref. | 75.5 | 79.3 | 12.4 | 98.8 |
| WC Z-score | 0.77 (0.69-0.84) | <0.001 | 64.2 | 81.2 | 11.8 | 98.3 |
| WHtR Z-score | 0.85 (0.80-0.90) | 0.616 | 92.5 | 63.3 | 8.9 | 99.5 |
| **CH** |  |  |  |  |  |  |
| BMI | 0.85 (0.73-0.98) | Ref. | 76.5 | 92.7 | 11.6 | 99.7 |
| WC | 0.82 (0.69-0.95) | 0.069 | 76.5 | 87.3 | 7.0 | 99.7 |
| WHtR | 0.86 (0.76-0.96) | 0.889 | 82.4 | 84.9 | 6.4 | 99.7 |
| BMI Z-score | 0.86 (0.75-0.98) | Ref. | 88.2 | 84.0 | 6.4 | 99.8 |
| WC Z-score | 0.85 (0.76-0.94) | 0.660 | 82.4 | 88.5 | 8.2 | 99.8 |
| WHtR Z-score | 0.88 (0.80-0.97) | 0.615 | 82.4 | 89.8 | 9.2 | 99.8 |

LVH, left ventricular hypertrophy; CR, concentric remodeling; EH, eccentric hypertrophy; CH, concentric hypertrophy; BMI, body mass index; WC, waist circumference; WHtR, waist-to-height ratio; AUC, area under the operating characteristic curve; *CI*, confidence interval; PPV, positive predictive value; NPV, negative predictive value.

^*^Comparisons of AUCs with BMI as the referent.

**Table S5. Characteristics of study participants by sex**

| **Characteristics** | **Total (n=1492)** | **Boys (n=793)** | **Girls (n=699)** | ***P* value^*^** |
| --- | --- | --- | --- | --- |
| Age, years | 8.90 (1.51) | 8.92 (1.52) | 8.88 (1.51) | 0.935 |
| BMI, kg/m ^2^ | 18.20 (3.45) | 18.71 (3.63) | 17.61 (3.15) | <0.001 |
| WC, cm | 62.98 (9.80) | 64.94 (10.31) | 60.76 (8.68) | <0.001 |
| WHtR | 0.46 (0.06) | 0.47 (0.06) | 0.45 (0.05) | <0.001 |
| BMI Z-score | 0.00 (1.00) | 0.00 (1.00) | 0.00 (1.00) | 0.969 |
| WC Z-score | 0.00 (1.00) | 0.00 (0.99) | 0.00 (1.00) | 0.996 |
| WHtR Z-score | 0.00 (1.00) | 0.00 (0.99) | 0.00 (1.00) | 0.976 |
| SBP, mmHg | 106.34 (9.20) | 107.18 (8.97) | 105.39 (9.37) | <0.001 |
| DBP, mmHg | 63.62 (6.68) | 63.45 (6.57) | 63.82 (6.80) | 0.276 |
| SBP Z-score | 0.00 (1.00) | 0.00 (1.00) | 0.00 (1.00) | 0.995 |
| DBP Z-score | 0.00 (1.00) | 0.00 (1.00) | 0.00 (0.99) | 0.937 |
| LVMI, g/m^2.7^ | 28.43 (4.79) | 29.70 (4.97) | 27.00 (4.14) | <0.001 |
| RWT, mm | 3.07 (0.20) | 3.09 (0.21) | 3.04 (0.18) | <0.001 |
| BMI-obese | 327 (21.9) | 192 (24.2) | 135 (19.3) | 0.023 |
| WC-obese | 470 (31.5) | 261 (32.9) | 209 (29.9) | 0.211 |
| WHtR-obese | 363 (24.3) | 244 (30.8) | 119 (17.0) | <0.001 |
| LVH | 146 (9.8) | 78 (9.8) | 68 (9.7) | 0.944 |
| High RWT | 155 (10.4) | 83 (10.5) | 72 (10.3) | 0.916 |
| Sleep duration <9 hours/day | 239 (16.0) | 116 (14.6) | 123 (17.6) | 0.119 |
| Screen time > 2 hours/day | 70 (4.7) | 37 (4.7) | 33 (4.7) | 0.960 |
| Physical activity time < 1 hour/day | 859 (57.6) | 454 (57.3) | 405 (57.9) | 0.788 |
| Intake of vegetable/fruit < 5 servings/day | 1214 (81.4) | 669 (84.4) | 545 (78.0) | 0.002 |
| Intake of soft drink ≥ 1 time/week | 91 (6.1) | 62 (7.8) | 29 (4.2) | 0.003 |
| ^*^Differences in characteristics between boys and girls were assessed using t test or chi-square test.  Continuous variables are expressed as means (standard deviations) and categorical variables as numbers (%).  BMI, body mass index; WC, waist circumference; WHtR, waist-to-height ratio; SBP, systolic blood pressure; DBP, diastolic blood pressure; LVMI, left ventricular mass index; RWT, relative wall thickness; LVH, left ventricular hypertrophy. | | | | |
